# Supplementary material for: Dynamics of labor and capital in AI vs. non-AI industries: A two-industry model analysis
Source: PLoS One. 2024 Jan 24;19(1):e0295150. doi: 10.1371/journal.pone.0295150 (PMC10807838; doi:10.1371/journal.pone.0295150)
Supplement: S1 Appendix — (DOCX) [file pone.0295150.s001.docx]

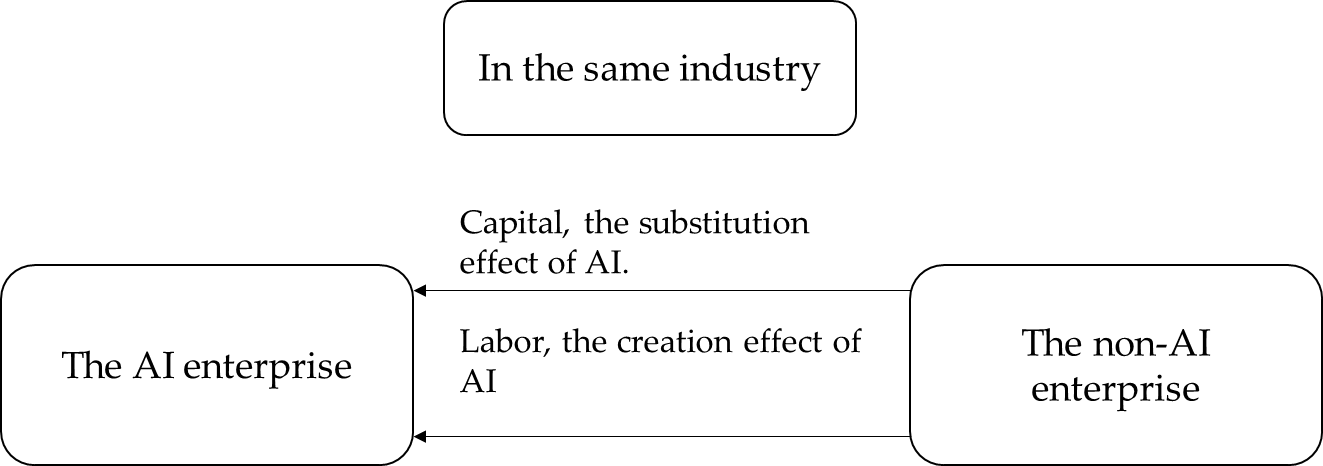


S1 Fig. In the same industry, capital flows between the AI enterprise and the non-AI enterprise.


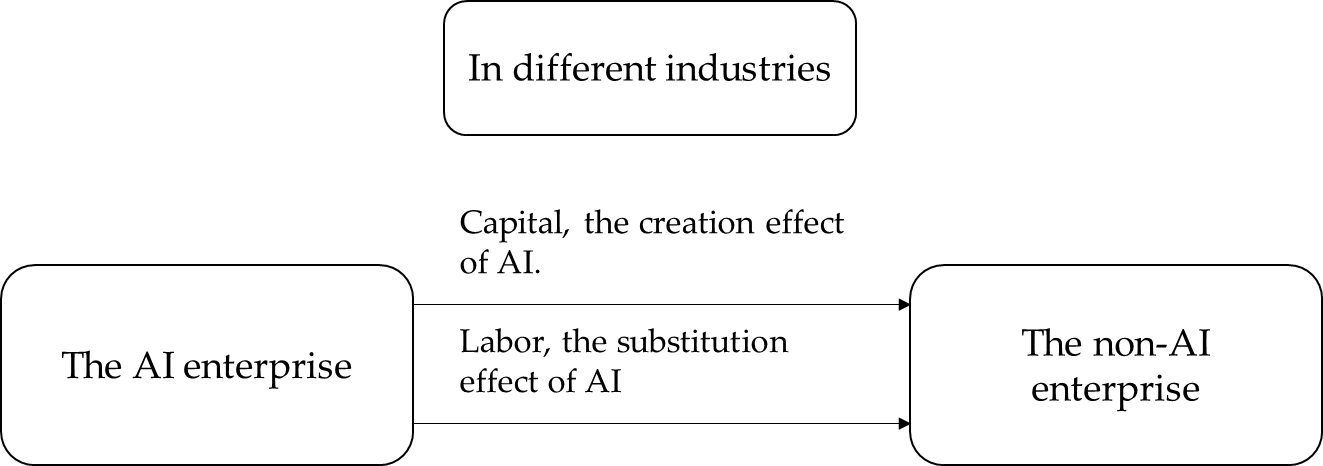


S2 Fig. In different industries, capital flows between the AI enterprise and the non-AI enterprise.


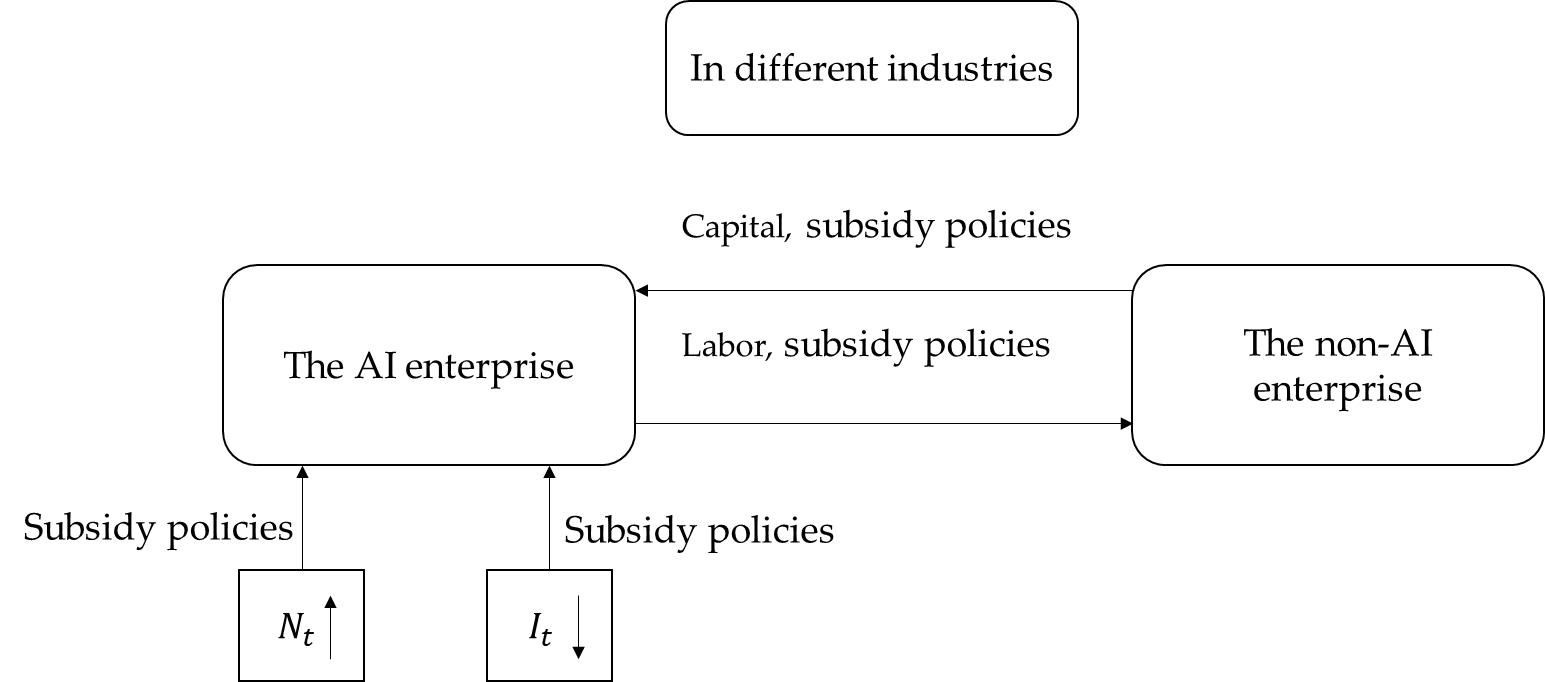


S3 Fig. The impact of subsidy policies on capital and labor mobility


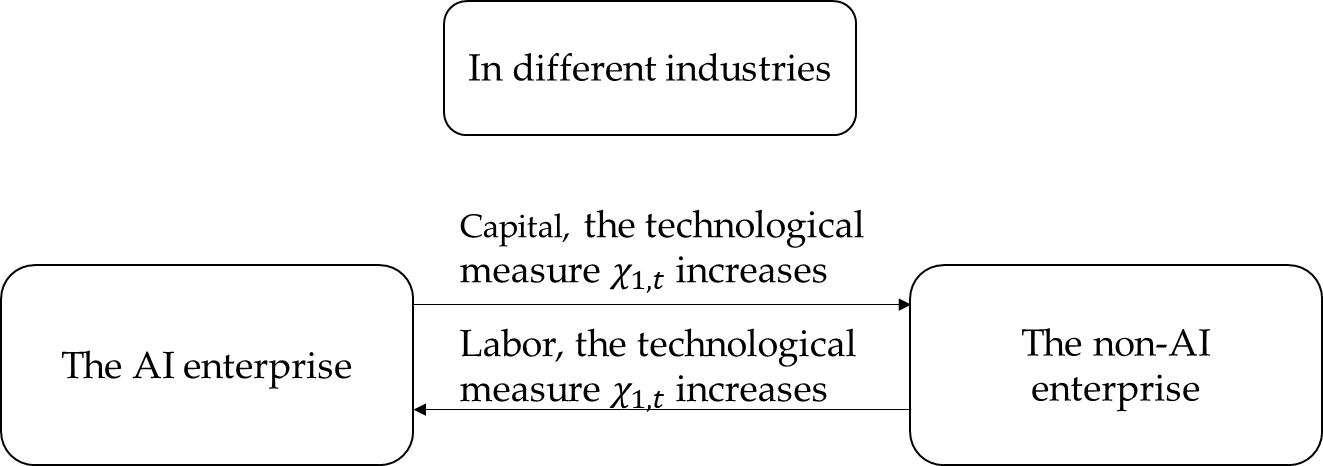


S4 Fig. When technological measures $\chi_{1,t}$ increase, capital and labor mobility between AI and non-AI enterprises.


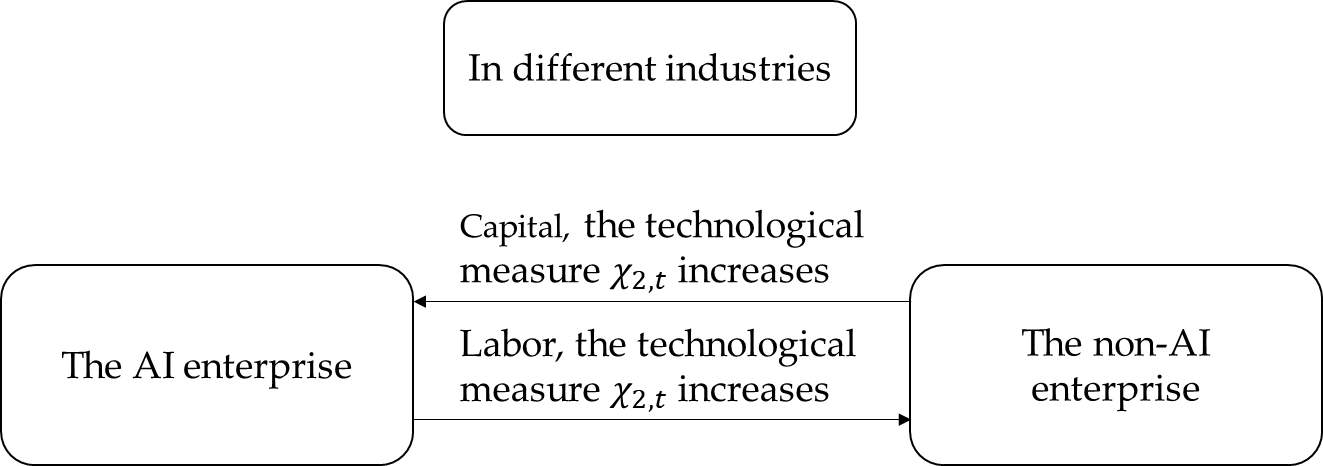


S5 Fig. When technological measures $\chi_{2,t}$ increase, capital and labor mobility between AI and non-AI enterprises.

S6 Fig. The impact of AI on two enterprises in the same industry with the same change range of $N_{t}$ and $I_{t}$

S7 Fig. Impact of AI on different industries with the same change range of $N_{t}$ and $I_{t}$.

S8 Fig. The impact of AI on enterprises in the same industry with $N_{t}$ unchanged and$I_{t}$ increasing

S9 Fig. The impact of AI on enterprises in different industries as $N_{t}$ remains unchanged and $I_{t}$ becomes larger.

S10 Fig. $I_{t}$ remains unchanged, $N_{t}$ becomes larger, and the impact of AI in the same industry.

S11 Fig. The impact of AI on different industries with $I_{t}$ unchanged and $N_{t}$ increasing.

S12 Fig. Economic impact of government subsidies policy

S13 Fig. Unemployment rate under government subsidy policy

S14 Fig. The economic impact of intelligent production by enterprises in both industries

**Appendix:**

**1. Proof of Proposition 1**

By (29), (31)

$\frac{\partial{\ln\omega}_{t}}{\partial I_{t}}=-\frac{1}{N_{t}-I_{t}}+\frac{\varepsilon-1}{\varepsilon}\cdot\frac{\partial(\ln Y_{1,t})}{\partial I_{t}}$, $\frac{\partial{\ln\omega}_{t}}{\partial N_{t}}=\frac{1}{N_{t}-I_{t}}+\frac{\varepsilon-1}{\varepsilon}\cdot\frac{\partial\left( \ln Y_{1,t} \right)}{\partial N_{t}}.$

Where $-\frac{1}{N_{t}-I_{t}}$ is the substitution effect, $\frac{\partial(\ln Y_{1,t})}{\partial I_{t}}$and $\frac{\partial\left( \ln Y_{1,t} \right)}{\partial N_{t}}$ are the productivity

effect.

$\frac{\partial\ln v_{t}}{\partial I_{t}}=\frac{\varepsilon-1}{\varepsilon}\cdot\frac{\partial(\ln Y_{1,t})}{\partial I_{t}}-\frac{2[\frac{1}{2}-(N_{t}-I_{t})]}{(N_{t}-I_{t})(I_{t}-N_{t}+1)}$

$\frac{\partial\ln v_{t}}{\partial N_{t}}=\frac{\varepsilon-1}{\varepsilon}\cdot\frac{\partial\left( \ln Y_{1,t} \right)}{\partial N_{t}}+\frac{2[\frac{1}{2}-(N_{t}-I_{t})]}{(N_{t}-I_{t})(I_{t}-N_{t}+1)}$

When $dI_{t}=dN_{t}$,

$d(\ln\omega_{t})=d(\ln v_{t})=\frac{\varepsilon-1}{\varepsilon}[\frac{\partial(\ln Y_{1,t})}{\partial N_{t}}+\frac{\partial(\ln Y_{1,t})}{\partial I_{t}}]dN_{t}$

Obtained by (14), (15)

$\frac{\partial(\ln Y_{1,t})}{\partial I_{t}}>0,\frac{\partial(\ln Y_{1,t})}{\partial N_{t}}>0$ and $0<N_{t}-I_{t}<\frac{1}{2}$

The share of Enterprise 1 in total social output is

$\alpha_{1,t}=\frac{p_{1,t}Y_{1},t}{Y_{t}}=\frac{\gamma Y_{1,t}^{\frac{\varepsilon-1}{\varepsilon}}}{\gamma Y_{1,t}^{\frac{\varepsilon-1}{\varepsilon}}+(\text{1-}\gamma)Y_{2,t}^{\frac{\varepsilon-1}{\varepsilon}}}=\frac{1}{1+\frac{1-\gamma}{\gamma}(\frac{Y_{2,t}}{Y_{1,t}})^{\frac{\varepsilon-1}{\varepsilon}}}$

The share of Enterprise 2 in total social output is

$\alpha_{2,t}=\frac{p_{2,t}Y_{2,t}}{Y_{t}}=\frac{(\text{1-}\gamma)Y_{2,t}^{\frac{\varepsilon-1}{\varepsilon}}}{\gamma Y_{1,t}^{\frac{\varepsilon-1}{\varepsilon}}+(\text{1-}\gamma)Y_{2,t}^{\frac{\varepsilon-1}{\varepsilon}}}=\frac{1}{1+\frac{\gamma}{\text{1-}\gamma}(\frac{Y_{1,t}}{Y_{2,t}})^{\frac{\varepsilon-1}{\varepsilon}}}$

(i)When$\varepsilon>1$，$\frac{\partial{\ln\omega}_{t}}{\partial N_{t}}>0,\frac{\partial{\ln v}_{t}}{\partial N_{t}}>0$；$\frac{\partial\ln\omega_{t}}{\partial I_{t}}$，$\frac{\partial\ln v_{t}}{\partial I_{t}}$ symbol is uncertain，

$d(\ln\omega_{t})=d(\ln v_{t})>0$，$\frac{\partial\alpha_{1,t}}{\partial N_{t}}=\frac{\partial\alpha_{1,t}}{\partial Y_{1,t}}\frac{\partial Y_{1,t}}{\partial N_{t}}>0$，$\frac{\partial\alpha_{2,t}}{\partial N_{t}}=\frac{\partial\alpha_{2,t}}{\partial Y_{2,t}}\frac{\partial Y_{2,t}}{\partial N_{t}}<0$,

$\frac{\partial\alpha_{1,t}}{\partial I_{t}}=\frac{\partial\alpha_{1,t}}{\partial Y_{1,t}}\frac{\partial Y_{1,t}}{\partial I_{t}}>0$，$\frac{\partial\alpha_{2,t}}{\partial I_{t}}=\frac{\partial\alpha_{2,t}}{\partial Y_{2,t}}\frac{\partial Y_{2,t}}{\partial I_{t}}<0.$

(ii) When$0<\varepsilon<1$，$\frac{\partial{\ln\omega}_{t}}{\partial I_{t}}<0$，$\frac{\partial{\ln v}_{t}}{\partial I_{t}}<0$；$\frac{\partial{\ln\omega}_{t}}{\partial N_{t}}$，$\frac{\partial{\ln v}_{t}}{\partial I_{t}}$ symbol is uncertain，

$d({\ln\omega}_{t})=d({\ln v}_{t})<0$，$\frac{\partial\alpha_{1,t}}{\partial N_{t}}=\frac{\partial\alpha_{1,t}}{\partial Y_{1,t}}\frac{\partial Y_{1,t}}{\partial N_{t}}<0$，$\frac{\partial\alpha_{2,t}}{\partial N_{t}}=\frac{\partial\alpha_{2,t}}{\partial Y_{2,t}}\frac{\partial Y_{2,t}}{\partial N_{t}}>0$,

$\frac{\partial\alpha_{1,t}}{\partial I_{t}}=\frac{\partial\alpha_{1,t}}{\partial Y_{1,t}}\frac{\partial Y_{1,t}}{\partial I_{t}}<0$，$\frac{\partial\alpha_{2,t}}{\partial I_{t}}=\frac{\partial\alpha_{2,t}}{\partial Y_{2,t}}\frac{\partial Y_{2,t}}{\partial I_{t}}>0$.

**2. Proof of Proposition 2:**

From (34)-(37), the following formula can be obtained:

$$\frac{\partial x_{k,t}}{\partial N_{t}}=\frac{1}{{(k_{t}+1)}^{2}}\cdot\frac{\partial k_{t}}{\partial N_{t}},\frac{\partial x_{k,t}}{\partial I_{t}}=\frac{1}{{(k_{t}+1)}^{2}}\cdot\frac{\partial k_{t}}{\partial I_{t}}$$

$$\frac{\partial x_{l,t}}{\partial N_{t}}=\frac{1}{{(l_{t}+1)}^{2}}\cdot\frac{\partial l_{t}}{\partial N_{t}},\frac{\partial x_{l,t}}{\partial I_{t}}=\frac{1}{{(l_{t}+1)}^{2}}\cdot\frac{\partial l_{t}}{\partial I_{t}}$$

By (34), (35)

$\frac{\partial\ln l_{t}}{\partial I_{t}}=-\frac{1}{N_{t}-I_{t}}+\frac{\varepsilon-1}{\varepsilon}\cdot\frac{\partial(\ln Y_{1,t})}{\partial I_{t}}$，$\frac{\partial\ln l_{t}}{\partial N_{t}}=\frac{1}{N_{t}-I_{t}}+\frac{\varepsilon-1}{\varepsilon}\cdot\frac{\partial\left( \ln Y_{1,t} \right)}{\partial N_{t}}$

$\frac{\partial\ln k_{t}}{\partial I_{t}}=\frac{\varepsilon-1}{\varepsilon}\cdot\frac{\partial\left( \ln Y_{1,t} \right)}{\partial I_{t}}+\frac{1}{I_{t}-N_{t}+1}$

$\frac{\partial\ln k_{t}}{\partial N_{t}}=\frac{\varepsilon-1}{\varepsilon}\cdot\frac{\partial(\ln Y_{1,t})}{\partial I_{t}}-\frac{1}{I_{t}-N_{t}+1}$

$dI_{t}=dN_{t}$，

$d(\ln l_{t})=d(\ln k_{t})=\frac{\varepsilon-1}{\varepsilon}[\frac{\partial(\ln Y_{1,t})}{\partial N_{t}}+\frac{\partial(\ln Y_{1,t})}{\partial I_{t}}]dN_{t}$

When $\varepsilon>1$, the sign of $\frac{\partial\ln l_{t}}{\partial I_{t}}$ and $\frac{\partial x_{l,t}}{\partial I_{t}}$ are uncertain, $\frac{\partial x_{k,t}}{\partial I_{t}}>0,\frac{\partial\ln k_{t}}{\partial I_{t}}>0.$

$\frac{\partial\ln l_{t}}{\partial N_{t}}>0,\frac{\partial x_{l,t}}{\partial N_{t}}>0$, the sign of $\frac{\partial\ln k_{t}}{\partial N_{t}}$ and $\frac{\partial x_{k,t}}{\partial N_{t}}$ are uncertain.$d\left( \ln l_{t} \right)=d\left( \ln k_{t} \right)>0$.

When $0<\varepsilon<1$, $\frac{\partial\ln l_{t}}{\partial I_{t}}<0$ and $\frac{\partial x_{l,t}}{\partial I_{t}}<0$, the sign of $\frac{\partial x_{k,t}}{\partial I_{t}} and \frac{\partial\ln k_{t}}{\partial I_{t}}$ are uncertain.

The sign of $\frac{\partial\ln l_{t}}{\partial N_{t}}$ and $\frac{\partial x_{l,t}}{\partial N_{t}}$ are uncertain, $\frac{\partial\ln k_{t}}{\partial N_{t}}<0,\frac{\partial x_{k,t}}{\partial N_{t}}<0$. $d\left( \ln l_{t} \right)=d\left( \ln k_{t} \right)>0.$

**3.** Proof **of Proposition 3:**

For (1) differential is available

$g=\frac{\gamma Y_{1}^{\frac{\varepsilon-1}{\varepsilon}}g_{1}+(\text{1-}\gamma)Y_{2}^{\frac{\varepsilon-1}{\varepsilon}}g_{2}}{\gamma Y_{1}^{\frac{\varepsilon-1}{\varepsilon}}+(\text{1-}\gamma)Y_{2}^{\frac{\varepsilon-1}{\varepsilon}}}$

when$\varepsilon>1$，$t\to\infty$，$g^{*}=\max\{g_{1}^{*},g_{2}^{*}\}$；when$0<\varepsilon<1$，$t\to\infty$，

$g^{*}=\min\{g_{1}^{*},g_{2}^{*}\}$.

Deriving the equation (7) with respect to time t,

$$g_{1}^{*}=\ln\frac{N}{N-1}+(I-N+1)m_{1}+(N-I)n_{1}+(\overset{\bullet}{I}-\overset{\bullet}{N})[\ln\frac{K_{1}}{L_{1}}-\ln(I-N+1)+\ln(N-I)]$$

And because $dN=dI$, so $\overset{\bullet}{Ndt}=\overset{\bullet}{I}dt$, $\overset{\bullet}{N}=\overset{\bullet}{I}$, substituting the above formula, we get (35). For (15), we get (36).

In equilibrium,$m_{1}=m_{2}=m,n_{1}=n_{2}=n$，so

$g_{1}^{*}-g_{2}^{*}=\ln\frac{N}{N-1}+(I-N+1)(m-n)-\sigma>0$.

**3. Proof of Proposition 4:**

Take the logarithm of (39) on both sides, constructor

$H=\ln(1-\tau_{1})+\ln k_{t}-\ln l_{t}-ln(1-e_{t})-\ln(I_{t}-N_{t}+1)+\ln(N_{t}-I_{t})-\ln(1- \alpha)+\ln\alpha$

Obtained by the implicit function theorem

$\frac{\partial k_{t}}{\partial\tau_{1}}=-\frac{\partial H/\partial\tau_{1}}{\partial H/\partial k_{t}}=\frac{k_{t}}{1-\tau_{1}}>0$，$\frac{\partial l_{t}}{\partial\tau_{1}}=-\frac{l_{t}}{1-\tau_{1}}<0$

$\frac{\partial I_{t}}{\partial\tau_{1}}=-\frac{(I_{t}-N_{t}+1)(N_{t}-I_{t})}{1-\tau_{1}}<0,\frac{\partial N_{t}}{\partial\tau_{1}}=\frac{(N_{t}-I_{t})(N_{t}-I_{t})}{1-\tau_{1}}>0$

$\frac{\partial e_{t}}{\partial\tau_{1}}=-\frac{\partial H/\partial\tau_{1}}{\partial H/\partial e_{t}}=\frac{{1-e}_{t}}{1-\tau_{1}}>0$

Obtained by (38),

$\frac{\partial(\ln y_{t})}{\partial\tau_{2}}=\frac{\varepsilon}{(1-\varepsilon)(1-\tau_{2})}>0$

From (32), $\tau_{1}$ is proportional to $\tau_{2}$, so the above conclusions are true for both $\tau_{1}$ and $\tau_{2}$.

**4. Proof of Proposition 6:**

(i) Obtained by (49), (51) and definition of technical measure

$v_{t}=\frac{\chi_{1,t}}{\chi_{2,t}}k_{t},l_{t}=\frac{\chi_{1,t}}{1-\chi_{1,t}}\cdot\frac{1-\chi_{2,t}}{\chi_{2,t}}k_{t}$

so

$\frac{\partial v_{t}}{\partial\chi_{1,t}}=\frac{k_{t}}{\chi_{2,t}}>0,\frac{\partial v_{t}}{\partial\chi_{2,t}}=-\frac{k_{t}\chi_{1,t}}{\chi_{2,t}^{2}}<0$

Take the logarithm of the two sides of $l_{t}$, constructor

$H=\ln l_{t}-\ln k_{t}+\ln(1-\chi_{1,t})-\ln\chi_{1,t}-\ln(1-\chi_{2,t})+\ln\chi_{2,t}$

Obtained by the implicit function theorem

$\frac{\partial l_{t}}{\partial\chi_{1,t}}=-\frac{\partial H/\partial\chi_{1,t}}{\partial H/\partial l_{t}}=\frac{l_{t}}{(1-\chi_{1,t})\chi_{1,t}}>0$，$\frac{\partial l_{t}}{\partial\chi_{2,t}}=-\frac{\frac{\partial H}{\partial\chi_{2,t}}}{\frac{\partial H}{\partial l_{t}}}=-\frac{l_{t}}{\left( 1-\chi_{2,t} \right)\chi_{2,t}}<0,$

$\frac{\partial k_{t}}{\partial\chi_{1,t}}=-\frac{\partial H/\partial\chi_{1,t}}{\partial H/\partial k_{t}}=-\frac{k_{t}}{(1-\chi_{1,t})\chi_{1,t}}<0$，$\frac{\partial k_{t}}{\partial\chi_{2,t}}=-\frac{\frac{\partial H}{\partial\chi_{2,t}}}{\frac{\partial H}{\partial k_{t}}}=\frac{k_{t}}{\left( 1-\chi_{2,t} \right)\chi_{2,t}}>0,$

$$\frac{\partial x_{l,t}}{\partial\chi_{1,t}}=\frac{\partial x_{l,t}}{\partial l_{t}}\cdot\frac{\partial l_{t}}{\partial\chi_{1,t}}=\frac{1}{\left( l_{t}+1 \right)^{2}}\cdot\frac{\partial l_{t}}{\partial\chi_{1,t}}>0,$$

$$\frac{\partial x_{l,t}}{\partial\chi_{2,t}}=\frac{\partial x_{l,t}}{\partial l_{t}}\cdot\frac{\partial l_{t}}{\partial\chi_{2,t}}=\frac{1}{\left( l_{t}+1 \right)^{2}}\cdot\frac{\partial l_{t}}{\partial\chi_{2,t}}<0,$$

$$\frac{\partial x_{k,t}}{\partial\chi_{1,t}}=\frac{\partial x_{k,t}}{\partial k_{t}}\cdot\frac{\partial k_{t}}{\partial\chi_{1,t}}=\frac{1}{\left( k_{t}+1 \right)^{2}}\cdot\frac{\partial k_{t}}{\partial\chi_{1,t}}<0,$$

$$\frac{\partial x_{k,t}}{\partial\chi_{2,t}}=\frac{\partial x_{l,t}}{\partial k_{t}}\cdot\frac{\partial k_{t}}{\partial\chi_{2,t}}=\frac{1}{\left( k_{t}+1 \right)^{2}}\cdot\frac{\partial k_{t}}{\partial\chi_{2,t}}>0.$$

（ii）when$d\chi_{1,t}=d\chi_{2,t}$,

$$dl_{t}=\frac{\partial l_{t}}{\partial\chi_{1,t}}d\chi_{1,t}+\frac{\partial l_{t}}{\partial\chi_{2,t}}d\chi_{2,t}=\frac{(\chi_{2,t}-\chi_{1,t})(1-\chi_{1,t}-\chi_{2,t})}{(1-\chi_{1,t})\chi_{1,t}(1-\chi_{2,t})\chi_{2,t}}l_{t}d\chi_{1,t}$$

$$dk_{t}=\frac{\partial k_{t}}{\partial\chi_{1,t}}d\chi_{1,t}+\frac{\partial k_{t}}{\partial\chi_{2,t}}d\chi_{2,t}=-\frac{(\chi_{2,t}-\chi_{1,t})(1-\chi_{1,t}-\chi_{2,t})}{(1-\chi_{1,t})\chi_{1,t}(1-\chi_{2,t})\chi_{2,t}}k_{t}d\chi_{1,t}$$

$$dx_{l,t}=\frac{1}{\left( l_{t}+1 \right)^{2}}dl_{t},dx_{k,t}=\frac{1}{\left( k_{t}+1 \right)^{2}}dk_{t}$$

$dv_{t}=\frac{\partial v_{t}}{\partial\chi_{1,t}}d\chi_{1,t}+\frac{\partial v_{t}}{\partial\chi_{2,t}}d\chi_{2,t}=\frac{\chi_{2,t}-\chi_{1,t}}{\chi_{2,t}^{2}}k_{t}d\chi_{1,t}$

(iii) Because $\frac{\gamma_{l_{1}}}{\gamma_{k_{1}}}>\frac{x_{1,t}}{1-x_{1,t}},\frac{\gamma_{l_{2}}}{\gamma_{k_{2}}}>\frac{x_{2,t}}{1-x_{2,t}}$*,*

Thus $\frac{\partial lnA_{1,t}}{\partial x_{1,t}}=ln\frac{\gamma_{l_{1}}(1-x_{1,t})}{\gamma_{k_{1}}x_{1,t}}>0,\frac{\partial lnA_{2,t}}{\partial x_{2,t}}=ln\frac{\gamma_{l_{2}}(1-x_{2,t})}{\gamma_{k_{2}}x_{2,t}}>0$.

$\frac{\partial\alpha_{L,t}}{\partial x_{1,t}}=\frac{{(\frac{Y_{1,t}}{Y_{2,t}})}^{\frac{\varepsilon-1}{\varepsilon}}}{{(\frac{Y_{1,t}}{Y_{2,t}})}^{\frac{\varepsilon-1}{\varepsilon}}+\frac{1-\gamma}{\gamma}}>0,\frac{\partial\alpha_{L,t}}{\partial x_{2,t}}=\frac{1}{{(\frac{Y_{1,t}}{Y_{2,t}})}^{\frac{\varepsilon-1}{\varepsilon}}+\frac{1-\gamma}{\gamma}}>0$

$\frac{\partial\alpha_{k,t}}{\partial x_{1,t}}=-\frac{\left( \frac{Y_{1,t}}{Y_{2,t}} \right)^{\frac{\varepsilon-1}{\varepsilon}}}{\left( \frac{Y_{1,t}}{Y_{2,t}} \right)^{\frac{\varepsilon-1}{\varepsilon}}+\frac{1-\gamma}{\gamma}}<0,\frac{\partial\alpha_{k,t}}{\partial x_{2,t}}=-\frac{1}{\left( \frac{Y_{1,t}}{Y_{2,t}} \right)^{\frac{\varepsilon-1}{\varepsilon}}+\frac{1-\gamma}{\gamma}}<0$
