# Supplementary material for: Dynamics of labor and capital in AI vs. non-AI industries: A two-industry model analysis
Source: PLoS One. 2024 Jan 24;19(1):e0295150. doi: 10.1371/journal.pone.0295150 (PMC10807838; doi:10.1371/journal.pone.0295150)
Supplement: Attachment — Submitted filename: Response to Reviewers.docx [file pone.0295150.s003.docx]

Dynamics of labor and capital in AI vs. Non-AI industries: A two-industry model analysis

I would like to thank the reviewers and editorial staff for their good and pertinent comments. This article has been revised as required, and the revised contents will be replied one by one as follows. The red text is the original expert opinion, and the black text is the reply opinion.

Reviewers' comments:

Reviewer's Responses to Questions

Comments to the Author

1. Is the manuscript technically sound, and do the data support the conclusions?

The manuscript must describe a technically sound piece of scientific research with data that supports the conclusions. Experiments must have been conducted rigorously, with appropriate controls, replication, and sample sizes. The conclusions must be drawn appropriately based on the data presented.

Reviewer #1: Yes

Reviewer #2: Partly

Reply: I have carefully revised this part of the paper, detailing the sources of data in the paper and attaching the corresponding data.

2. Has the statistical analysis been performed appropriately and rigorously?

Reviewer #1: I Don't Know

Reviewer #2: N/A

Reply: This article generates corresponding data through numerical simulation and does not use empirical analysis methods. The minimum data set for numerical simulation is provided based on China's actual economic conditions.

3. Have the authors made all data underlying the findings in their manuscript fully available?

The PLOS Data policy requires authors to make all data underlying the findings described in their manuscript fully available without restriction, with rare exception (please refer to the Data Availability Statement in the manuscript PDF file). The data should be provided as part of the manuscript or its supporting information, or deposited to a public repository. For example, in addition to summary statistics, the data points behind means, medians and variance measures should be available. If there are restrictions on publicly sharing data—e.g. participant privacy or use of data from a third party—those must be specified.

Reviewer #1: Yes

Reviewer #2: No

Reply: Thanks to the experts for their valuable advice. I will provide the corresponding data sets in the form of appendix material.

4. Is the manuscript presented in an intelligible fashion and written in standard English?

PLOS ONE does not copyedit accepted manuscripts, so the language in submitted articles must be clear, correct, and unambiguous. Any typographical or grammatical errors should be corrected at revision, so please note any specific errors here.

Reviewer #1: Yes

Reviewer #2: No

Reply: Thanks to the experts for their valuable opinions, I carefully read the entire text and made grammatical revisions.

5. Review Comments to the Author

Please use the space provided to explain your answers to the questions above. You may also include additional comments for the author, including concerns about dual publication, research ethics, or publication ethics. (Please upload your review as an attachment if it exceeds 20,000 characters)

Reviewer #1: The paper presents a two-industry model that examines the dynamics between AI and non-AI enterprises. While the topic is timely and holds significant relevance to the current trends in the global economy, there are various aspects of the manuscript that require revision and clarification. Below are the suggestions:

Clarity of Model Assumptions:

The assumptions underlying your two-industry model are not clearly laid out in the abstract. The model's soundness can only be ascertained with a clear understanding of these assumptions. Kindly elucidate.

Reply: Thanks to the experts for their valuable opinions, which helped me understand a lot. There are too many contents in the model assumptions. We elaborate on them in the Basic Assumptions section, which are listed below: The entire society is categorized into the primary, secondary, and tertiary sectors, with the share of the primary sector increasing in China, the share of the secondary sector remaining stable, and the share of the tertiary sector continuously rising. This article does not consider the primary sector and focuses solely on the secondary and tertiary sectors. Therefore, this article employs a two-industry model for theoretical analysis. The AI enterprise is one that extensively utilizes AI technologies and automation equipment in its production and operations, such as a manufacturing enterprise employing industrial robots. Conversely, the non-AI enterprise is one that relies on traditional mate-rial production methods and is typically a labor-intensive enterprise.

Methodological Approach:

The methodology used to derive conclusions from the model should be detailed in the main body. For instance, how are you quantifying the 'consumption effect'? How is the flow of labor and capital between enterprises measured or predicted?

Reply: Thanks to the experts for their valuable opinions, which helped me understand the problems of the paper. Your opinions have improved my understanding of the problems. The Task-based model will be employed to analyze the impact of artificial intelligence on industrial transformation and upgrading. Subsequently, this paper will utilize data from China to conduct numerical simulations aimed at validating the propositions.

The statement of the consumer effect was not quite accurate, so I changed it to the price effect in the paper. The price effect is calculated through formula (3) and is listed as follows:

The relative price $p_{t}$ is defined as

$p_{t}\equiv\frac{p_{1,t}}{p_{2,t}}=\frac{\partial Y_{t}/\partial Y_{1,t}}{\partial Y_{t}/\partial Y_{2,t}}=\frac{\gamma}{1-\gamma}\cdot(\frac{Y_{1,t}}{Y_{2,t}})^{-\frac{1}{\varepsilon}}$, (3)

According to equation (3), this article represents the price effect and market demand effect through variations in $p_{t}$. When $p_{t}$ increases, the price of products from the AI enterprise rises more rapidly compared to that of the non-AI enterprise. The increase in product price by the AI enterprise stimulates an expansion in its production, which represents the market demand effect. Conversely, when $p_{t}$ decreases, the product price for the non-AI enterprise increases at a faster rate, prompting the non-AI enterprise to increase its output.

To investigate the issue of labor and capital mobility, this article assumes that the allocation of capital and labor in enterprise 1 is governed by the following function:

${x_{k,t}=\frac{K_{1,t}}{K_{t}}, x}_{l,t}=\frac{L_{1,t}}{L_{t}} .$	(31)

Hence, the capital and labor proportions in enterprise 2 are $1-x_{k,t}$ and $1-x_{l,t}$ respectively. When $x_{k,t}$ and $x_{l,t}$ change, there will be a flow of capital and labor between enterprise 1 and enterprise 2. We measure the changes in capital and labor through $x_{k,t}$ and $x_{l,t}$. The process of reallocating production factors also reflects the process of industrial structural transformation and upgrading.

Combined with equation (30) with (10), (11), (17), the functional form for the capital and labor proportions of enterprise 1 is as follows:

$x_{k,t}=\frac{(N_{t}-I_{t})\gamma Y_{1,t}^{\frac{\varepsilon-1}{\varepsilon}}}{\left( N_{t}-I_{t} \right)\gamma Y_{1,t}^{\frac{\varepsilon-1}{\varepsilon}}+(1-\alpha)(1-\gamma)Y_{2,t}^{\frac{\varepsilon-1}{\varepsilon}}},$	(32)	$x_{l,t}=\frac{(N_{t}-I_{t})\gamma Y_{1,t}^{\frac{\varepsilon-1}{\varepsilon}}}{\left( N_{t}-I_{t} \right)\gamma Y_{1,t}^{\frac{\varepsilon-1}{\varepsilon}}+(1-\alpha)(1-\gamma)Y_{2,t}^{\frac{\varepsilon-1}{\varepsilon}}}$. (33)

Taxation and Policy Implications:

The abstract suggests a governmental intervention via taxation to promote the improvement of production efficiency. However, such a recommendation demands a deeper empirical or simulation-based analysis. The consequences of such taxation, especially in the context of potential unemployment, need a rigorous analysis.

Reply: Thanks to the experts for their valuable advice. Your comments hit the nail on the head about the problem with the paper, and I learned a lot from it. In reality, governments levy taxes on both the AI and non-AI enterprises. However, when the government adopts subsidy policies, it essentially equates to providing tax relief to the AI enterprise. This subsidy policy fundamentally involves utilizing tax revenue collected from non-AI enterprise to support the AI enterprise. To simplify the analysis, this paper assumes that the government imposes taxes on the non-AI enterprise and subsequently utilizes these tax proceeds to subsidize the AI enterprise. Another rationale for employing the aforementioned analysis is that artificial intelligence exhibits spillover effects. This implies that the non-AI enterprise benefits from positive externalities arising from the innovation and development of the AI enterprise. Hence, the government can rectify this situation by imposing corrective taxes.

In this section, to examine the impact of subsidy policies on employment, we define the unemployment rate as $e_{t}$. Given that artificial intelligence is expected to replace a significant portion of the workforce, we assume that the unemployed individuals originate from the pool of labor employed by intelligent enterprises. Initially, the labor force of intelligent enterprise 1 is denoted as $L_{1,t}$, and the number of unemployed individuals is $e_{t}L_{1,t}$. As a result, the workforce of enterprise 1 subsequently becomes $(1-e_{t})L_{1,t}$.

The AI enterprise receives subsidies and pursues profit maximization.

$\max_{K_{1,t},L_{1,t}}\pi_{1,t}=p_{1,t}Y_{1,t}-W_{1,t}{\left( 1-e_{t} \right)L}_{1,t}-\left( 1-\tau_{1} \right)R_{1,t}K_{1,t},$	(44)

Based on theoretical analysis, I have found that increasing the subsidy rate will lead to a higher unemployment rate. This is because as the subsidy rate rises, enterprises will increasingly adopt artificial intelligence for production. This results in a reduction in the labor force employed by enterprises, thus leading to an increase in the unemployment rate.

This article also discusses the unemployment rate under government subsidy policies, as shown in Figure 8. Numerical simulation found that as the subsidy rate increases, enterprises will adopt more intelligent devices. The productivity effect reduces the use of labor and increases unemployment. Consumers have not increased their demand for manufacturing products. Under the suppression of price effects, the intelligentization process of enterprises will stop, so the unemployment rate will not continue to increase.

**Fig 8. Unemployment rate under government subsidy policy**

Clarification on 'Consumption Effect':

The term 'consumption effect' is introduced without a clear definition or context. Please explain the mechanism behind this effect, especially in the context of your model.

Reply: Thanks to the experts for their valuable advice. The expression of the consumption effect is not accurate and should be changed to the price effect. According to equation (3), this article represents the price effect through variations in $p_{t}$. When $p_{t}$ increases, the price of products from the AI enterprise rises more rapidly compared to that of the non-AI enterprise. The increase in product price by the AI enterprise stimulates an expansion in its production, which represents the market demand effect. Conversely, when $p_{t}$ decreases, the product price for the non-AI enterprise increases at a faster rate, prompting the non-AI enterprise to increase its output.

Relevance of 'Labor and Capital Share':

The conclusion mentions the tendency of labor and capital share to converge. While this is an interesting result, its relevance and implications in the broader context of the paper should be clarified.

Reply: Thank you for the valuable insights, your perspective is very insightful. I realize that the paper needs to provide a clearer elucidation of the labor share and capital share. The modifications are listed below.

The expressions for the total labor share and capital share are as follows:

$\alpha_{L,t}=\frac{\omega_{1,t}L_{1,t}+\omega_{2,t}L_{2,t}}{Y_{t}}=\frac{x_{1,t}{(\frac{Y_{1,t}}{Y_{2,t}})}^{\frac{\varepsilon-1}{\varepsilon}}+x_{2,t}}{{(\frac{Y_{1,t}}{Y_{2,t}})}^{\frac{\varepsilon-1}{\varepsilon}}+\frac{1-\gamma}{\gamma}}$	(64)

$\alpha_{K,t}=\frac{R_{1,t}K_{1,t}+R_{2,t}K_{2,t}}{Y_{t}}=1-\frac{x_{1,t}{(\frac{Y_{1,t}}{Y_{2,t}})}^{\frac{\varepsilon-1}{\varepsilon}}+x_{2,t}}{{(\frac{Y_{1,t}}{Y_{2,t}})}^{\frac{\varepsilon-1}{\varepsilon}}+\frac{1-\gamma}{\gamma}}$	(65)

Proposition 6. (iii) When enterprises adopt artificial intelligence technology that is biased towards improving labor productivity, increasing technical measurement will improve the level of artificial intelligence technology, thereby increasing enterprise output. When enterprises increase their technical measures, the total labor share will increase and the total capital share will decrease.

Technological advancements increase R&D value for firms by expanding labor tasks, fostering a positive link between technological measures and R&D value. This shifts firms toward labor over capital. For example, if Firm 1 improves its technological measures, it attracts labor from Firm 2 while reducing its capital demand, causing capital to move to Firm 2. If both firms share the same technological advancement, initial technological measures decide labor and capital flow. If Firm 2's measure surpasses Firm 1's, Firm 1's higher R&D value leads to increased labor demand and reduced capital demand, moving capital from Firm 1 to Firm 2. When both firms possess identical technological measures, labor and capital demands balance out, resulting in no net flow. This implies that artificial intelligence development promotes convergence, where firms with differing initial technological measures gradually approach similar labor and capital allocation levels.

Data Source and Real-world Applications:

The model would greatly benefit from real-world data or case studies that support your findings. If the conclusions are purely theoretical, the paper should also provide suggestions on how to test these conclusions empirically.

Reply: Thanks for your valuable advice. This paper primarily investigates the development status of two sectors: manufacturing and services, as they currently contribute significantly to China's GDP. According to data from the National Bureau of Statistics of China, in 2010, the value-added by the secondary sector accounted for 46.5% of GDP, the tertiary sector accounted for 44.2%, while the primary sector accounted for only 9.3%. Furthermore, over time, the contribution of the primary sector has been decreasing annually and in 2021, it accounted for just 7.3%. According to data from the National Bureau of Statistics, in 2010, the population engaged in manufacturing was 218 million, while the population engaged in the service sector was 263 million. This paper primarily examines the impact of artificial intelligence on the economy, disregarding demographic factors. Therefore, we assume a population growth rate of 0. In sensitivity analysis, a population growth rate of -0.3% is set, but its impact on the results can be considered negligible.

Reviewer #2: Title and Scope:

Consider refining the title for a clearer indication of the primary focus and outcomes of the study. Perhaps: "Dynamics of Labor and Capital in AI vs. Non-AI Industries: A Two-Industry Model Analysis".

Reply: Thank you for your valuable advice. In the paper, I reflect the transformation and upgrading of enterprises through the flow of capital and labor. Your suggestion is better. I will adopt your suggestion to revise the thesis title.

Elaborate on the significance and implications of the identified imbalance in AI development across industries in the introduction.

Reply: Thank you for your valuable advice. I have made major revisions in the introduction, as follows:

In China, the development of artificial intelligence (AI) applications shows regional and industry disparities [8,9]. Major cities like Beijing, Shanghai, and Shenzhen lead in AI advancement due to resources and technology advantages, while smaller cities and rural areas lag behind due to tech, talent, and funding challenges. AI adoption also varies by industry. Finance, e-commerce, and the internet rapidly embrace AI with substantial data and digital foundations, boosting efficiency and innovation. However, traditional sectors like manufacturing progress more slowly, facing transformation challenges [10].

These differences in AI adoption leads to disparities in production efficiency and innovation, favoring AI-advanced industries [11]. Labor-intensive sectors are slower to adopt AI, risking missed efficiency and innovation gains [12]. Unequal AI deployment affects the labor market. Some industries automate tasks, reducing job opportunities. Yet, AI creates new roles in AI development and data science, often requiring advanced skills and education, potentially worsening skill disparities [13]. These disparities can exacerbate social inequality, favoring those in automated industries with better economic opportunities. Conversely, workers in low-skilled or traditional sectors may face unemployment or stagnant wages, widening wealth and opportunity gaps, especially in AI-intensive industries [14].

Model Assumptions:

Clearly state and justify the underlying assumptions for your two-industry model. How are "AI enterprises" and "non-AI enterprises" explicitly defined? Discuss the real-world relevance and validity of these assumptions. Which industries or scenarios might this model be most applicable to?

Reply: Thank you for your valuable advice. You read it very carefully and gave me a deeper understanding of writing a paper. I made the corresponding modifications, listed below:

The entire society is categorized into the primary, secondary, and tertiary sectors, with the share of the primary sector increasing in China, the share of the secondary sector remaining stable, and the share of the tertiary sector continuously rising. This article does not consider the primary sector and focuses solely on the secondary and tertiary sectors. Therefore, this article employs a two-industry model for theoretical analysis. The AI enterprise is one that extensively utilizes AI technologies and automation equipment in its production and operations, such as a manufacturing enterprise employing industrial robots. Conversely, the non-AI enterprise is one that relies on traditional mate-rial production methods and is typically a labor-intensive enterprise.

Model Dynamics:

Provide a detailed explanation and illustration (perhaps a flowchart or diagram) of how labor and capital move between the AI and non-AI enterprise under different conditions.

Reply: Thank you for your valuable opinions. Your opinions are very pertinent. Drawing the corresponding flow diagram really helps to understand the paper. The flow diagrams of Propositions 1~2 are as follows:

Fig 1. In the same industry, capital flows between the AI enterprise and the non-AI enterprise.

Fig 2. In different industries, capital flows between the AI enterprise and the non-AI enterprise.

In Figure 1, the substitution effect of artificial intelligence leads to capital flowing from the non-AI enterprise to the AI enterprise, while the productivity effect of AI leads to labor moving from the non-AI enterprise to the AI enterprise. A similar explanation can also be applied to Figure 2.

Fig 3. The impact of subsidy policies on capital and labor mobility

Figure 3 illustrates the impact of subsidy policies on the mobility of capital and labor. In the long term, this policy results in an AI enterprise increasing its use of intelligent capital for production, thereby reducing its reliance on labor. As a consequence, capital flows from a non-AI enterprise to the AI enterprise, while labor moves from the AI enterprise to a non-AI enterprise.

Fig 4. When technological measures $\chi_{1,t}$ increase, capital and labor mobility between AI and non-AI enterprises.

Fig 5. When technological measures $\chi_{2,t}$ increase, capital and labor mobility between AI and non-AI enterprises.

In Figure 4 and Figure 5, when technological measure χ_(1,t) increases, capital flows from an AI enterprise to a non-AI enterprise, while labor moves from a non-AI enterprise to an AI enterprise. When technological measure χ_(2,t) increases, capital and labor experience the opposite flow.

Clarify the "consumption effect" that benefits the non-AI enterprise more. Is this a well-established concept, or is it novel to this paper? Please provide adequate references or theoretical underpinnings.

Reply: Thank you for the valuable input. The expression 'consumer effect' is not accurate, and the correct terminology should be 'price effect.' The definition of the price effect is derived from the following paper:

Acemoglu D, Guerrieri V. Capital deepening and nonbalanced economic growth[J]. Journal of political Economy, 2008, 116(3): 467-498.

The final product price is standardized to 1. The product price produced by the AI enterprise 1 is $p_{1,t}$, and the product price produced by the non-AI enterprise is $p_{2,t}$,so that

$p_{1,t}=\frac{\partial Y_{t}}{\partial Y_{1,t}}=\gamma{(\frac{Y_{1,t}}{Y_{t}})}^{-\frac{1}{\varepsilon}},p_{2,t}=\frac{\partial Y_{t}}{\partial Y_{2,t}}=\gamma{(\frac{Y_{2,t}}{Y_{t}})}^{-\frac{1}{\varepsilon}}$	(2)

The relative price $p_{t}$ is defined as

$p_{t}\equiv\frac{p_{1,t}}{p_{2,t}}=\frac{\partial Y_{t}/\partial Y_{1,t}}{\partial Y_{t}/\partial Y_{2,t}}=\frac{\gamma}{1-\gamma}\cdot(\frac{Y_{1,t}}{Y_{2,t}})^{-\frac{1}{\varepsilon}}$, (3)

and

$[\gamma^{\varepsilon}p_{1,t}^{1-\varepsilon}+(1-\gamma)p_{2,t}^{1-\varepsilon}]^{\frac{1}{1-\varepsilon}}=1$. (4)

According to equation (3), this article represents the price effect through variations in $p_{t}$. When $p_{t}$ increases, the price of products from the AI enterprise rises more rapidly compared to that of the non-AI enterprise. The increase in product price by the AI enterprise stimulates an expansion in its production. Conversely, when $p_{t}$ decreases, the product price for the non-AI enterprise increases at a faster rate, prompting the non-AI enterprise to increase its output [47].

Government Intervention:

Discuss the rationale and potential consequences of taxing non-AI enterprises while subsidizing AI enterprises. Would this approach be feasible in practice?

Reply: Thanks for the valuable advice, your insights are very profound. In reality, governments levy taxes on both the AI and non-AI enterprises. However, when the government adopts subsidy policies, it essentially equates to providing tax relief to the AI enterprise. This subsidy policy fundamentally involves utilizing tax revenue collected from non-AI enterprise to support the AI enterprise. To simplify the analysis, this paper assumes that the government imposes taxes on the non-AI enterprise and subsequently utilizes these tax proceeds to subsidize the AI enterprise. Another rationale for employing the aforementioned analysis is that artificial intelligence exhibits spillover effects. This implies that the non-AI enterprise benefits from positive externalities arising from the innovation and development of the AI enterprise. Hence, the government can rectify this situation by imposing corrective taxes.

Delve deeper into the ethical considerations of such policies, especially when considering their potential to exacerbate unemployment and further inhibit non-AI enterprise development.

Reply: Thank you for your valuable advice. Your insights are very profound. We should indeed pay more attention to the workforce. Proposition 4 points out that subsidy policies will indeed increase the unemployment rate. In the long term, as the level of automation increases, there may be a significant risk of widespread unemployment. Some workers may need to acquire new skills to adapt to evolving job requirements, which can be a time-consuming and resource-intensive process. Governments will need to formulate appropriate policies to assist these workers in retraining and securing reemployment opportunities.

Short-Term Effects:

The paper mentions that taxation has only a short-term effect on AI development. Please provide evidence or theoretical reasoning behind this claim.

Reply: Thank you for your valuable opinions. We have already proved it in the paper, as follows:

The AI enterprise only considers the impact of subsidies on the automation process from its own perspective. If there is no subsidy, at the critical point $I$, AI enterprises use capital and labor at the same cost. ie $\frac{R_{1,t}}{\gamma_{k_{1}}(I_{t})}=\frac{W_{1,t}}{\gamma_{l_{1}}(I_{t})}$, and the deformation is$\frac{\gamma_{l_{1}}(I_{t})}{\gamma_{k_{1}}(I_{t})}=\frac{W_{1,t}}{R_{1,t}}$. After the government subsidizes, the equilibrium point changes to $I_{\tau_{1}}$, that is, $\frac{(1-\tau_{1})R_{1,t}}{\gamma_{k_{1}}(I_{\tau_{1}})}=\frac{W_{1,t}}{\gamma_{l_{1}}(I_{\tau_{1}})}$, and the deformation is $\frac{\gamma_{l_{1}}(I_{\tau_{1}})}{\gamma_{k_{1}}(I_{\tau_{1}})}=\frac{W_{1,t}}{(1-\tau_{1})R_{1,t}}$, so $\frac{\gamma_{l_{1}}(I_{t})}{\gamma_{k_{1}}(I_{t})}<\frac{\gamma_{l_{1}}(I_{\tau_{1}})}{\gamma_{k_{1}}(I_{\tau_{1}})}$. Since $\gamma_{l_{1}}(i)/\gamma_{k_{1}}(i)$ is monotonically increasing on $I$, so $I_{t}<I_{\tau_{1}}$, that is, the government's subsidy policy for the AI enterprise will indeed encourage enterprises to buy automation equipment and accelerate machine substitution. Of course, this is only from the production point of view. The above promotion of enterprises to improve the level of AI application through subsidies is called the productivity effect.

After theoretical derivation, Proposition 4 points out that subsidy policies will promote artificial intelligence to generate new tasks and delay the automation process. Therefore, subsidy policies only have short-term effects.

Consider examining the potential long-term impacts of these policy suggestions, both positive and negative.

Reply: Thank you for your valuable opinions. I have made the necessary modifications, as follows:

The positive effects of subsidy policies include:

(i)Stimulating intelligent enterprises to procure more intelligent equipment, thereby accelerating the process of automation and enhancing production efficiency. This aids in im-proving product quality and reducing production costs, subsequently increasing the competitiveness of enterprises. (ii)Significantly boosting the output of intelligent enterprises, allowing them to offer more high-quality products and services in both domestic and inter-national markets. This is likely to increase export volumes, promote GDP growth, and inject vitality into economic development. (iii)Subsidy policies encourage non-intelligent enterprises to actively adopt intelligent strategies, raising the overall level of automation in society. This, in turn, promotes industrial upgrading, creates more high-skilled jobs, offers greater development opportunities for workers, and contributes to economic growth and sustainability across society.

Negative impacts of subsidy policies include:

(i)Some non-intelligent enterprises may find it challenging to transition to automation, particularly in sectors such as communication and courier services. These enterprises may face competitive pressures, leading to business contraction or closure, which could affect employment and local economies. (ii)Subsidy policies do not guarantee a continuous and uninterrupted pace of automation. Over time, the rate of automation adoption may gradually decelerate. This may result in reduced investment demands for intelligent enterprises and impact the development of related industrial supply chains. (iii)In the long term, as the level of automation increases, there may be a significant risk of widespread unemployment. Some workers may need to acquire new skills to adapt to evolving job requirements, which can be a time-consuming and resource-intensive process. Governments will need to formulate appropriate policies to assist these workers in retraining and securing reemployment opportunities.

Unemployment Concerns:

Address the unemployment risks in more detail. What industries or worker profiles might be most vulnerable? How significant is the risk?

Reply: Thank you for your valuable opinions. I have made the necessary modifications, as follows: In this section, to examine the impact of subsidy policies on employment, we define the unemployment rate as $e_{t}$. Given that artificial intelligence is expected to replace a significant portion of the workforce, we assume that the unemployed individuals originate from the pool of labor employed by intelligent enterprises. Initially, the labor force of intelligent enterprise 1 is denoted as $L_{1,t}$, and the number of unemployed individuals is $e_{t}L_{1,t}$. As a result, the workforce of enterprise 1 subsequently becomes $(1-e_{t})L_{1,t}$. Based on Frey & Osborne (2017)[48], the probability of worker unemployment risk is denoted as $r_{s,t}=\frac{1}{1+exp(-I_{t})}$. The probability of unemployment risk, as expressed in the aforementioned form, ensures both $0 < r_{s,t} < 1$ and a positive correlation between the unemployment risk probability $r_{s,t}$ and the level of automation $I_{t}$. It should be explicitly noted that the probability of unemployment risk $r_{s,t}$ pertains to a priori probability, whereas the unemployment rate $e_{t}$ pertains to a posteriori probability, and these two are not the same.

Equilibrium Analysis:

The conclusion suggests that labor and capital shares will equalize when both industries use AI. Provide a thorough analysis of how and why this equilibrium is reached.

Reply: Thank you for your valuable opinions. Changes in technological measures will lead to adjustments in an enterprise's artificial intelligence technologies, thereby resulting in varying levels of development across different departments. Such technological shifts will impact the enterprise's output, ultimately causing fluctuations in product prices and subsequently prompting the flow of capital and labor. I have made the necessary modifications, as follows:

From (10), (11), (52), (53), (54)

$l_{t}=\frac{N_{t}-I_{t}}{I_{t}-N_{t}+1}\cdot\frac{J_{t}-M_{t}+1}{M_{t}-J_{t}}k_{t},$	(55)

$\frac{x_{l,t}}{1-x_{l,t}}=\frac{N_{t}-I_{t}}{I_{t}-N_{t}+1}\cdot\frac{J_{t}-M_{t}+1}{M_{t}-J_{t}}\frac{x_{k,t}}{1-x_{k,t}}.$	(56)

Where $l_{t}\equiv\frac{L_{1,t}}{L_{2,t}},k_{t}\equiv\frac{K_{1,t}}{K_{2,t}},{x_{k,t}=\frac{K_{1,t}}{K_{t}}, x}_{l,t}=\frac{L_{1,t}}{L_{t}}$.

Similar to the analysis above

$V_{1,t}=\frac{\pi_{k_{1,t}}}{r(t)}=(N_{t}-I_{t})K_{1,t},V_{2,t}=\frac{\pi_{k_{2,t}}}{r(t)}=(M_{t}-J_{t})K_{2,t}$, (57)

$v_{t}\equiv\frac{V_{1,t}}{V_{2,t}}=\frac{N_{t}-I_{t}}{M_{t}-J_{t}}k_{t}$. (58)

From (56),

$\frac{dlnx_{k,t}}{dln(A_{1,t}/A_{2,t})}=\frac{(1-x_{k,t})(\varepsilon-1)}{1+(\varepsilon-1)(M_{t}-J_{t}-N_{t}+I_{t})(x_{k,t}-x_{l,t})}<0$	(59)

$\frac{dlnx_{l,t}}{dln(A_{1,t}/A_{2,t})}=\frac{(1-x_{l,t})(\varepsilon-1)}{1+(\varepsilon-1)(M_{t}-J_{t}-N_{t}+I_{t})(x_{k,t}-x_{l,t})}<0$	(60)

$\frac{dlnx_{k,t}}{dln(K)}=\frac{(1-x_{k,t})(M_{t}-J_{t}-N_{t}+I_{t})(\varepsilon-1)}{1+(\varepsilon-1)(M_{t}-J_{t}-N_{t}+I_{t})(x_{k,t}-x_{l,t})}<0$	(61)

$\frac{dlnx_{l,t}}{dln(K)}=\frac{(1-x_{l,t})(M_{t}-J_{t}-N_{t}+I_{t})(\varepsilon-1)}{1+(\varepsilon-1)(M_{t}-J_{t}-N_{t}+I_{t})(x_{k,t}-x_{l,t})}<0$	(62)

**Proposition 5.** (i) When Enterprise 1 enhances its artificial intelligence technology, capital and labor flow from Enterprise 1 to Enterprise 2. When Enterprise 2 improves its artificial intelligence technology, capital and labor flow from Enterprise 2 to Enterprise 1.

(ii) When the total capital stock increases, capital and labor shift from Enterprise 1 to Enterprise 2.

Changes in technological measures will lead to adjustments in an enterprise's artificial intelligence technologies, thereby resulting in varying levels of development across different departments. Such technological shifts will impact the enterprise's output, ultimately causing fluctuations in product prices and subsequently prompting the flow of capital and labor. Using the manufacturing and service industries as examples, when the manufacturing industry enhances its production efficiency through the utilization of artificial intelligence technologies, it experiences an increase in output, subsequently leading to lower product prices. However, due to relatively stable price demand, individuals do not purchase more manufacturing products simply because they have become more affordable. This results in a reduction in the prices of manufacturing products and a relative increase in the prices of products in the service industry. The price effect then steers capital and labor from the manufacturing industry to the service industry.

Conversely, when the service industry improves its efficiency through the adoption of artificial intelligence technologies, capital and labor flow from the service industry to the manufacturing industry. An increase in the overall capital stock prompts capital and labor to migrate from industries with higher technological sophistication to those with lower technological advancement.

This transformation underscores the fact that societal development depends not only on its areas of expertise but also on its areas of relative deficiency. When society's production factors are relatively abundant, the price effect guides production factors from industries with higher technological proficiency to those with lower technological sophistication, thus promoting balanced development across the entire society.

Discuss the societal and economic implications of such an equilibrium. What does it mean for industry growth, innovation, and wealth distribution?

Reply: Thank you for your valuable advice. Your insights are very profound. I have made the following modifications, as listed below:

In order to simplify the analysis, this article assumes that artificial intelligence technology has the same productivity on different tasks, that is, $\gamma_{l_{1}}=\gamma_{l_{1}}\left( i \right),\gamma_{k_{1}}=\gamma_{k_{1}}\left( i \right),\gamma_{l_{2}}=\gamma_{l_{2}}\left( i \right),\gamma_{k_{2}}=\gamma_{k_{2}}\left( i \right)$. When the technical measure increases, the enterprise's labor share will increase, that is, the increase in technical measure tends to increase labor productivity. In order to reflect the above characteristics, this article assumes

$\frac{\gamma_{l_{1}}}{\gamma_{k_{1}}}>\frac{x_{1,t}}{1-x_{1,t}},\frac{\gamma_{l_{2}}}{\gamma_{k_{2}}}>\frac{x_{2,t}}{1-x_{2,t}}$	(63)

The expressions for the total labor share and capital share are as follows:

$\alpha_{L,t}=\frac{\omega_{1,t}L_{1,t}+\omega_{2,t}L_{2,t}}{Y_{t}}=\frac{x_{1,t}{(\frac{Y_{1,t}}{Y_{2,t}})}^{\frac{\varepsilon-1}{\varepsilon}}+x_{2,t}}{{(\frac{Y_{1,t}}{Y_{2,t}})}^{\frac{\varepsilon-1}{\varepsilon}}+\frac{1-\gamma}{\gamma}}$	(64)

$\alpha_{K,t}=\frac{R_{1,t}K_{1,t}+R_{2,t}K_{2,t}}{Y_{t}}=1-\frac{x_{1,t}{(\frac{Y_{1,t}}{Y_{2,t}})}^{\frac{\varepsilon-1}{\varepsilon}}+x_{2,t}}{{(\frac{Y_{1,t}}{Y_{2,t}})}^{\frac{\varepsilon-1}{\varepsilon}}+\frac{1-\gamma}{\gamma}}$	(65)

**Proposition 6.** (i) When the technological measure $\chi_{1,t}$ increases, the research and development value $V_{1,t}$ of firm 1 increases at a faster rate, leading to an increase in the labor proportion and a decrease in the capital proportion of firm 1. Conversely, when the technological measure $\chi_{2,t}$ increases, the research and development value $V_{2,t}$ of firm 2 increases at a faster rate, resulting in a decrease in the labor proportion and an increase in the capital proportion of firm 1.

(ii) When $d\chi_{1,t}=d\chi_{2,t}$，if$\chi_{2,t}>\chi_{1,t}$，then $dv_{t}>0,dx_{l,t}>0,dx_{k,t}<0$.If $\chi_{2,t}=\chi_{1,t}$，then $dv_{t}=dx_{l,t}=dx_{k,t}=0$. If $\chi_{2,t}<\chi_{1,t}$，then $dv_{t}<0,dx_{l,t}<0,dx_{k,t}>0.$

(iii) When enterprises adopt artificial intelligence technology that is biased towards improving labor productivity, increasing technical measurement will improve the level of artificial intelligence technology, thereby increasing enterprise output. When enterprises increase their technical measures, the total labor share will increase and the total capital share will decrease.

The economic intuitions underlying Proposition 6 can be summarized as follows:

(1) An increase in technological measures leads to an increase in the R&D value for firms because it increases the number of labor tasks. This implies a positive relationship between technological measures and R&D value. When technological measures improve, firms will rely more on labor rather than capital. For example, an increase in technological measures for Firm 1 will increase the demand for labor, attracting labor from Firm 2. Meanwhile, Firm 1's demand for capital decreases, leading to a flow of capital toward Firm 2.

(2) When both firms experience the same level of technological advancement, the initial size of their technological measures determines the flow of labor and capital. If Firm 2's technological measure is greater than that of Firm 1, then the R&D value of Firm 1 will be greater than that of Firm 2. Since R&D value tends to favor labor, Firm 1 will have a greater demand for labor, attracting labor from Firm 2. At the same time, Firm 1's demand for capital decreases, causing capital to flow from Firm 1 to Firm 2.

(3) When both firms have the same technological measures, the demand for labor and capital is equal for both Firm 1 and Firm 2, leading to a halt in the flow of labor and capital. This suggests that the development of artificial intelligence technology tends to lead to convergence, where firms with different initial technological measures gradually approach equal levels of labor and capital shares.

Literature Review:

Ensure that the paper incorporates a comprehensive review of existing literature, touching upon AI adoption rates across industries, economic implications of AI, and policy recommendations from other studies.

Reply: Thank you for your valuable feedback. I have made significant revisions to the introduction, and I have also updated the references. Please refer to the introduction section of the paper for specific details.

How does this study differentiate or expand upon existing research?

Reply: Thanks for your valuable opinions, I have sorted out the innovations of this paper. The possible innovations of this article are as follows: Firstly, this article expands the theoretical framework of the task-based model, expanding the theoretical research of artificial intelligence from a single department to two departments. This article points out the impact of artificial intelligence on capital and labor mobility in the same industry and different industries, thereby clarifying the impact of artificial intelligence on worker skills. Secondly, this study points out the short-term and long-term effects of government subsidy policies on artificial intelligence, which has reference significance for the government to implement public policies on artificial intelligence. Thirdly, this study points out that if artificial intelligence is widely applied throughout the industry in the future, technological measures will bring changes in capital and labor shares. This has important reference significance for the future development of artificial intelligence.

Limitations:

Clearly state the limitations of the two-industry model. Are there external factors or industry-specific nuances that the model doesn't account for?

Reply: Thank you for your valuable opinions. I have made the necessary modifications, as follows:

While the two-industry model presented in this paper has yielded certain research findings, it still exhibits several limitations, providing fruitful directions for more in-depth investigation in the future:

(i) The paper exclusively considers a closed economic system, neglecting the broader in-fluence of other nations. Future research endeavors can extend this model to open economies to gain a better understanding of the economic implications of disparate artificial intelligence development in developed and developing countries.

(ii) This paper primarily examines the manufacturing and service sectors from a macroeconomic perspective, without delving deeply into the intricacies of these sectors. Future studies could conduct more detailed analyses of specific industries, aligning with re-al-world scenarios, to unveil additional nuances and insights.

(iii) The focus of this paper on subsidy policies represents only a fraction of public poli-cy considerations. Subsequent research can explore a broader spectrum of public policies, such as increased investments in public education and novel infrastructure, to comprehensively comprehend their impact on the field of artificial intelligence.

Recommendations:

Beyond taxation, offer alternative policy recommendations or corporate strategies that could alleviate the identified challenges or optimize the benefits of AI adoption across industries.

Reply: Thank you for your valuable opinions. I have made the necessary modifications, as follows:

The policy implications of this article are as follows.

(i) Strengthen technology research and development to promote the balanced development of artificial intelligence among various industries. Within the same industry, the AI enterprise will occupy the dominant position of the department and will stimulate other companies to accelerate the process of artificial intelligence. However, due to the existence of technological externalities between different industries, the non-AI industry lacks the incentive to innovate. In order to promote the increase of social growth rate, the government can invest funds in the research and development of non-artificial intelligence industries, encourage scientific research institutions to develop technologies that have not realized the automation industry.

(ii) The government can adopt appropriate tax policies to make transfer payments. For example, in the initial stage, in order to stimulate the development of artificial intelligence in all industries, the government can subsidize artificial intelligence enterprises. To help enterprises accelerate the process of artificial intelligence, the government can subsidize enterprises that purchase artificial automation equipment to help enterprises reduce costs.

(iii) Although subsidies can accelerate artificial intelligence, they only have short-term effects. In the long run, in order to promote sustainable and stable economic development, governments and enterprises must adhere to the path of independent research and development.

(iv) The government can offer incentives and rewards to encourage businesses to engage in research and development (R&D) and innovation in the field of artificial intelligence. These incentives may encompass tax benefits, patent protection, and financial support for R&D, all aimed at stimulating corporate investments in technology and innovation.

(v) The government can foster collaboration and knowledge sharing among different industries to expedite the dissemination and application of technology. This can help low-er barriers to technology diffusion, promoting technology sharing and collaborative innovation.

(vi) The government can implement social welfare policies to alleviate the impact on individuals who lose their jobs due to technological transformations. This may include providing unemployment benefits, retraining opportunities, and career guidance to assist individuals in transitioning smoothly into new employment sectors.

These policy recommendations are designed to promote equitable development of artificial intelligence across diverse industries while ensuring sustainable economic growth and stability in society. Governments, businesses, and research institutions can collaborate to formulate a comprehensive policy framework that addresses the challenges and opportunities of the artificial intelligence era.

The following is the reply to the editor's opinion. The red part is the editor's opinion and the black part is my reply.

Journal Requirements:

When submitting your revision, we need you to address these additional requirements.

1. Please ensure that your manuscript meets PLOS ONE's style requirements, including those for file naming. The PLOS ONE style templates can be found at

https://journals.plos.org/plosone/s/file?id=wjVg/PLOSOne_formatting_sample_main_body.pdf and

https://journals.plos.org/plosone/s/file?id=ba62/PLOSOne_formatting_sample_title_authors_affiliations.pdf

Reply: Thanks to the editor for your valuable comments, I have carefully revised it according to the journal format.

2.Thank you for stating the following financial disclosure:

"This work was supported by the Ningbo Natural Science Foundation (2023J061), the Ningbo Philosophy and Social Science Foundation(G2023-2-41), Young Innovative Talents of Guang-dong Ordinary Colleges and Universities (Humanities and Social Sciences) (2021WONCX145), the Recycling and Remanufacturing Network Organization and Its Governance under the Sharing Economy of China under Grant (18BGL184), Zhejiang Provincial Natural Science Foundation of China under Grant (LY20G030025)."

Please state what role the funders took in the study. If the funders had no role, please state: ""The funders had no role in study design, data collection and analysis, decision to publish, or preparation of the manuscript.""

If this statement is not correct you must amend it as needed.

Please include this amended Role of Funder statement in your cover letter; we will change the online submission form on your behalf.

Reply: Thanks to the editor for his valuable comments, we have made corresponding adjustments to the fund.

Funding: This work was supported by Chinese National Funding of Social Sciences (23BJY134), the Ningbo Natural Science Foundation (2023J061), the Ningbo Philosophy and Social Science Foundation(G2023-2-41), Research Project Outcome of Zhejiang Provincial Federation of Social Sciences (2024N075), Ministry of Education Humanities and Social Sciences Research Youth Fund Project (22YJC790077), Young Innovative Talents of Guangdong Ordinary Colleges and Universities (Humanities and Social Sciences) (2021WONCX145), Zhejiang Provincial Natural Science Foundation of China (LY20G030025).

Among them, the Chinese National Funding of Social Sciences (23BJY134) funded the cost of printing and copying, the Ningbo Natural Science Foundation (2023J061) funded the cost of attending academic conferences, and the Ningbo Philosophy and Social Science Foundation (G2023-2-41) funded the literature Search costs, Zhejiang Provincial Federation of Social Sciences (2024N075) funded the cost of paper translation, The Ministry of Education Humanities and Social Sciences Research Youth Fund Project (22YJC790077) funded the cost of data collection. Young Innovative Talents of Guangdong Ordinary Colleges and Universities (Humanities and Social Sciences) (2021WONCX145) funded labor remuneration costs, Zhejiang Provincial Natural Science Foundation of China (LY20G030025). funded the cost of expert consultation.

The Recycling and Remanufacturing Network Organization and Its Governance under the Sharing Economy of China under Grant (18BGL184) had no role in study design, data collection and analysis, decision to publish, or preparation of the manuscript.

3. In your Data Availability statement, you have not specified where the minimal data set underlying the results described in your manuscript can be found. PLOS defines a study's minimal data set as the underlying data used to reach the conclusions drawn in the manuscript and any additional data required to replicate the reported study findings in their entirety. All PLOS journals require that the minimal data set be made fully available. For more information about our data policy, please see http://journals.plos.org/plosone/s/data-availability.

""Upon re-submitting your revised manuscript, please upload your study’s minimal underlying data set as either Supporting Information files or to a stable, public repository and include the relevant URLs, DOIs, or accession numbers within your revised cover letter. For a list of acceptable repositories, please see http://journals.plos.org/plosone/s/data-availability#loc-recommended-repositories. Any potentially identifying patient information must be fully anonymized.

Important: If there are ethical or legal restrictions to sharing your data publicly, please explain these restrictions in detail. Please see our guidelines for more information on what we consider unacceptable restrictions to publicly sharing data: http://journals.plos.org/plosone/s/data-availability#loc-unacceptable-data-access-restrictions. Note that it is not acceptable for the authors to be the sole named individuals responsible for ensuring data access.

We will update your Data Availability statement to reflect the information you provide in your cover letter.

Reply: Thanks to the editor for his valuable comments. The data is published in Figshare with the file name The minimum data set.zip. (https://figshare.com/account/home)

User name: [huangxu@nbufe.edu.cn](mailto:huangxu@nbufe.edu.cn) Password:Zcq616717@

4. We note that you have stated that you will provide repository information for your data at acceptance. Should your manuscript be accepted for publication, we will hold it until you provide the relevant accession numbers or DOIs necessary to access your data. If you wish to make changes to your Data Availability statement, please describe these changes in your cover letter and we will update your Data Availability statement to reflect the information you provide.

Reply: Thanks to the editor for his valuable comments. I have made corresponding modifications in the paper.
